# Immunomodulatory Properties of Vitamin D in the Intestinal and Respiratory Systems

**DOI:** 10.3390/nu15071696

**Published:** 2023-03-30

**Authors:** Fatheia N. Hamza, Sarah Daher, Hana M. A. Fakhoury, William B. Grant, Peter R. Kvietys, Khaled Al-Kattan

**Affiliations:** 1 College of Medicine, Alfaisal University, P.O. Box 50927, Riyadh 11533, Saudi Arabia; 2 Sunlight, Nutrition, and Health Research Center, P.O. Box 641603, San Francisco, CA 94164-1603, USA

**Keywords:** vitamin D, VDR signaling, antimicrobial peptides, innate immunity, inflammatory bowel disease (IBD), acute respiratory distress syndrome (ARDS), epithelial barrier, toll-like receptor (TLR)

## Abstract

Vitamin D plays a crucial role in modulating the innate immune response by interacting with its intracellular receptor, VDR. In this review, we address vitamin D/VDR signaling and how it contributes to the regulation of intestinal and respiratory microbiota. We additionally review some components of the innate immune system, such as the barrier function of the pulmonary and intestinal epithelial membranes and secretion of mucus, with their respective modulation by vitamin D. We also explore the mechanisms by which this vitamin D/VDR signaling mounts an antimicrobial response through the transduction of microbial signals and the production of antimicrobial peptides that constitute one of the body’s first lines of defense against pathogens. Additionally, we highlight the role of vitamin D in clinical diseases, namely inflammatory bowel disease and acute respiratory distress syndrome, where excessive inflammatory responses and dysbiosis are hallmarks. Increasing evidence suggests that vitamin D supplementation may have potentially beneficial effects on those diseases.

## 1. Introduction

The epithelial lining of both the gut (≈32 m^2^) and lung (≈70 m^2^) constitutes an extensive interface between the internal sterile milieu and the external microbe-laden environment [1,2]. Thus, a single layer of epithelial cells has the daunting task of assimilating essentials from the external environment while simultaneously minimizing microbial infection. The enterocytes of intestinal villi hydrolyze and absorb nutrients from ingested food, while the thin pneumocytes of the alveoli facilitate the diffusional uptake of oxygen. To minimize infection, the epithelium of both organs mounts an evolutionarily ancient system of host defense (innate immunity) [3]. Specifically, specialized epithelial cells secrete mucus and antimicrobial peptides (AMP) that serve as physical and chemical barriers that hinder ingested or inhaled microbes from accessing the epithelium [4,5,6,7]. Based on studies in the diploblastic holobiont, *Hydra*, it is posited these components of the innate immune response arose due to selection pressures encountered by the phylogenetic emergence of a mucosal epithelium within a microbe-laden habitat [8,9].

In the event microbes manage to traverse the mucus/AMP shield and approach the epithelium, resident phagocytes (e.g., macrophages) clear them from the affected area [3]. The specifics of phagocyte clearance differ between the lungs and the intestines. In the alveoli, mobile resident macrophages patrol the airspace and phagocytose any microbes encountered [10]. In the intestines, dense surface mucus and peristaltic shear forces preclude patrolling of the epithelial surface by resident macrophages. Instead, macrophages in the lamina propria can extend transepithelial dendrites to probe luminal contents and capture microbes [11,12]. The objective of macrophage phagocytosis and clearance of microbes is to neutralize the microbial threat while minimizing tissue inflammation and injury. Should recruitment of additional phagocytes (e.g., neutrophils) be required to aid in the clearance of the affected site, timely resolution of the inflammatory response and tissue injury are required to limit adverse systemic sequelae [13,14].

Vitamin D is an important modulator of the innate immune response to infection [15,16]. From an evolutionary perspective, the immunoregulatory function of vitamin D predates its role in bone homeostasis [17] and is currently present in invertebrates lacking an adaptive immune system [18]. Despite some divergent signaling pathways in vertebrates, salient mechanisms involved in vitamin D modulation of innate immune responses to infection are conserved remarkably [16]. 

Skin- or dietary-derived vitamin D is activated and inactivated by a series of hydroxylation reactions mediated by cytochrome P450 enzymes. In brief, hepatic CYP2R1 generates 25-hydroxyvitamin D [25(OH)D], which is the major circulating form of the vitamin, although it is inactive. A second hydroxylation by CYP27B1 converts 25D to the biologically active 1,25-dihydroxyvitamin D [1,25(OH)_2_D]. Vitamin D exerts its antimicrobial, anti-inflammatory, and epithelial-sparing functions by binding to the vitamin D receptor (VDR). Moreover, CYP24A1 is a strongly induced VDR target gene, which encodes an enzyme that mediates a physiological negative feedback loop by inactivating 1,25(OH)_2_D to an inactive trihydroxy form [1,24,25 (OH)_3_D]. VDR, along with the regulatory enzymes CYP27B1 and CYP24A1, are highly expressed in the diverse constituent cells of the gastrointestinal and respiratory systems (Table 1). VDR contains a ligand-binding domain (LBD) at the C-terminus and a DNA-binding domain allowing for the 1,25(OH)_2_D-activated VDR to act as a transcription factor [19]. VDR-induced transcription is usually mediated as a heterodimer with retinoid X receptor (RXR) interacting with vitamin D response elements (VDREs) on DNA. It is worth noting that VDR/RXR binding to a VDRE only occurs on genes whose transcription is activated, and not on repressed genes where the mechanisms of regulation are more heterogeneous. VDR can also exert rapid actions, which are independent of direct transcriptional events (e.g., protein-protein interactions), albeit specific mechanisms for non-genomic functions of VDR are less clear [20]. 

Excessive inflammatory response and loss of epithelial integrity are hallmarks of the clinical entities of inflammatory bowel disease (IBD) [41] and acute respiratory distress syndrome (ARDS) [42,43]. Both entities pose a risk of immunothrombosis [44], specifically a thrombogenic state driven by neutrophil extracellular traps (NETs) and potentially impacting remote organs/tissues [45,46]. A majority of IBD [47,48] and ARDS [49,50,51] patients are deemed vitamin D-deficient [25(OH)D < 20 ng/mL], and correction of vitamin D status has been advocated as an adjunct to conventional therapeutic regimens. However, prophylactic or interventional clinical studies of vitamin D supplementation on outcomes for either of these maladies, while promising, have not yielded definitive beneficial effects [26,35,48,50,52,53]. This could be due to confounding issues such as varying dosing regimens [50,53] individual responsiveness to supplementation [54], or the question of whether vitamin D deficiency in IBD patients is the cause or effect of the inflammatory response [26,53]. 

It is important to note that most vitamin D clinical trials have been conducted incorrectly since they are based on the guidelines for pharmaceutical drugs, not on nutrients [55]. In drug trials, the only source of the drug is in the trial and there is expected to be a linear dose-response relationship. For vitamin D, the guidelines for nutrient trials as outlined by Heaney in 2014 should be used [56]. This implies that the trials should be based on serum 25(OH)D concentrations, not the vitamin D dose. 

Furthermore, a study by Smolders et al. [57] proposed the concept of reverse causality, where acute inflammation leads to decreased levels of 25(OH)D in humans, as levels of circulating 25(OH)D were found to be at their lowest 2–3 h post-infusion of *E. coli*-derived lipopolysaccharide (LPS), which coincided with the release of proinflammatory cytokines such as tumor-necrosis factor (TNF)-α, IL-6, and IL-8 [57]. The implication of the Smolders et al. study [57] is that the serum 25(OH)D concentration at the time of hospital admission should not be used to determine the effect of the serum 25(OH)D concentration on the risk of infection. To more accurately measure the effect of vitamin D on the risk of infection, it is necessary to look at observational studies that measure serum 25(OH)D concentration at least two weeks prior to COVID-19. Such studies can provide a better understanding of the effect of vitamin D on COVID-19 and other infections. 

In this regard, a study conducted in Barcelona compared COVID-19 rates for patients who were prescribed either vitamin D_3_ or calcifediol [25(OH)D_2_] and achieved serum 25(OH)D levels > 30 ng/mL with those who had serum 25(OH)D levels < 20 ng/mL and were not prescribed any form of vitamin D [58]. The results showed that the former group had a significantly lower risk of COVID-19 than the latter group. A similar finding was reported for patients who received vitamin D prescriptions at the U.S. Department of Veterans Administration health care system [59]. These findings highlight the potential role of vitamin D in supporting immune function and protecting against respiratory infections. Similarly, a study by Dancer et al. found that vitamin D deficiency is prevalent in individuals who develop ARDS, and this deficiency appears to contribute to the development of the condition [60]. Moreover, Raftery et al. found that supplementing vitamin D to patients with IBD in remission helped maintain intestinal barrier integrity by preserving intestinal permeability. This highlights the crucial role of vitamin D in gut health [61]. Hence, the bulk of the experimental and clinical studies indicate that the antimicrobial, anti-inflammatory, and epithelial-sparing functions of vitamin D may be relevant to both IBD and ARDS.

Herein, we will review the innate immune response of the gut and lungs to infection as well as the potential sequelae of an excessive inflammatory response (immunothrombosis). The impact of vitamin D on innate immune pathways will be highlighted. To this end, the PubMed and Google Scholar databases were mined for relevant information derived from both experimental and, where applicable, clinical studies. A rationale for addressing commonalities of the innate immune response of the gut and lungs lies in their shared embryologic origin; salient features that are common to both will be presented. On the other hand, the gut and lungs are distinct anatomical and functional entities that preclude generalizations; the specifics of which will also be addressed. 

## 2. Microbiota of the Gut and Lungs

Both the intestines and lungs must contend with their resident microbiota and those encountered in the course of performing their respective functions. In healthy volunteers, seeding of both the gut and lung microbiota is via the common oropharyngeal pathway for swallowing and breathing. Predictably, the gastric microbial profile is like that of the mouth [62]. Of note, a significant fraction of the oral microbiota is also delivered to the lungs by micro-aspiration [62,63]. Microbes surviving gastric acidity are driven by peristaltic activity to the colon, the major habitat of the gut microbiota [4]. Microbes entering the bronchi are forced back via ciliary activity toward the larger airways and trachea, the major habitat of lung microbiota [64]. The resident microbial communities of both organs are not stable, but dynamic, with losses being replenished with new arrivals. Due to inter-individual variability, there is little consensus regarding the ideal microbial composition for either gut or lung homeostasis [64,65]. However, in healthy individuals, *Bacteroidetes* and *Firmicutes* are the predominant phyla of the local microbiota. *Proteobacteria* contribute only 5% and 15% of the microbial community of the gut and lungs, respectively [65,66]. Of note, the density of the colonic microbiota is orders of magnitude greater than that of the upper airways [64,67]. Some of the colonic microbes are expelled with feces while bronchial microbes are expectorated or swallowed. 

For the acquired and retained microbes, successful colonization of a niche is dependent on a favorable milieu with nutritional support. In health, the microbiota of both organs is successful in exploiting the respective niches and outcompeting any potential pathogenic microbes. The colonic lumen is oxygen-poor (pO_2_ < 10 mm Hg) [68] and favors anaerobes which can derive energy from the fermentation of undigested dietary (e.g., cellulose) or endogenous carbohydrates (e.g., mucin glycans) [26,69]. It is generally held that healthy microbiota preferentially ferment carbohydrates generating short-chain fatty acids (SCFAs), which benefit the host by providing an energy source and maintaining mucus secretion [70]. An outgrowth of mucolytic microbes is favored by dietary restriction of fiber and can predispose the colon to inflammation [71]. On the other hand, the airways are oxygen-rich (pO_2_ > 90 mm Hg) and aerobes would be favored [64]. Nutritional support for lung microbes is rather sparse, primarily host-derived (e.g., mucus), and believed to be dependent on the interactivity of its members [63,72,73]. The microbial pool of the lower airways is more transient than that of the colon and largely determined by transfer from and back to oropharyngeal secretions, with little contribution from in situ proliferation [74]. 

Alterations in the resident microbiota of the gut and lungs (dysbiosis) have been implicated in the clinical entities of IBD and ARDS. The dysbiosis of IBD is characterized by a loss of stability (extensive deviations over time), loss of diversity, a shift from obligate anaerobes to facultative aerobes (loss of beneficial effects of SCFA), as well as an outgrowth of pathobionts [28,75,76,77]. Approaches designed to reverse IBD dysbiosis (probiotics, fecal transplant) show some promise, albeit their effectiveness in clinical trials is short-lived and inconclusive [78,79,80]. An interesting complication of ARDS (referred to as “more of the gut in the lung”) is described for critically ill septic ARDS patients [81,82,83]. The microbial population in the lungs of ARDS patients can be enriched in gut-associated microbes (more so, in sepsis caused by extrapulmonary rather than intrapulmonary infections) [84]. Infection of the lungs by gut-associated microbes is attributed to either oropharyngeal aspiration or translocated from the gut [81]. Due to the inter-individual variability and a low-biomass pool, a specific microbial signature for ARDS is lacking [84]. Nonetheless, a probiotic approach has been explored in mice, yielding promising results [85]. 

## 3. Microbiota of the Gut and Lungs: Vitamin D/VDR Signaling

Vitamin D/VDR signaling has been implicated in the regulation of gut microbiota in health and disease (e.g., IBD) [26,29]. Studies in genetically manipulated murine models (e.g., VDR^−/−^, CYP27B1^−/−^) are in general agreement with human studies [26,29]. However, since prokaryotes do not have a VDR, a direct role of vitamin D/VDR signaling in the microbes is unlikely [86]. Rather, any impact of VDR signaling on the gut microbiota profile is attributed to host responses as borne out by either epithelial- or myeloid-specific VDR^−/−^ mice [87,88]. A similar case has been made for the impact of vitamin D/VDR signaling on the microbiota of the lungs [89]. 

## 4. Transduction of Microbial Signals to an Antimicrobial Response

A successful innate immune response to clear the infection depends on the rapid detection of microbes and the deployment of defense systems (e.g., mucus, AMPs, and phagocytosis). The epithelium of both the gut and lungs can detect microbial signals and transduce these signals into an appropriate effector response. Numerous molecular motifs derived from microbes/pathogens (microbe- or pathogen-associated molecular patterns and MAMPs or PAMPs) are detected by specific epithelial or macrophage pattern-recognition receptors (PRRs) [3,90,91,92]. 

PRRs are generally characterized as transmembrane or cytosolic. TLRs are transmembrane proteins expressed as homodimers or heterodimers on plasma or endosomal membranes [90,91]. TLRs comprise extracellular leucine-rich repeats (LRRs) that are responsible for microbial sensing, a membrane-spanning domain, and a cytoplasmic effector domain, which transduces PAMP signals into an antimicrobial response. Nucleotide-binding oligomerization domain leucine-rich repeats containing receptors (NLRs) are a major family of cytosolic PRRs, consisting of a C-terminal LLR sensor, a central nucleotide-binding oligomerization domain (NOD), and an N-terminal effector domain. NOD2 and NLRP3 are two major members of this family that form effector platforms by oligomerization referred to as “nodosomes” and “inflammasomes”, respectively [91,93]. While NOD2 activation and oligomerization are elicited by a specific entity, bacteria-derived muramyl dipeptide (MDP) [93], a myriad of entities (e.g., ROS, toxins, and cytokines) have been implicated in NLRP3 oligomerization, operating at priming and activating phases [94]. NOD2 activation results in an increase in transcriptional activity of NFκB, while the major outcome of NLRP3 inflammasome activation is the generation of active Il-1β. Interestingly, the colitis noted in NOD2^−/−^ mice is attributed to enhanced activation of NLRP3 inflammasome [95]. 

A major transcriptional pathway central to TLR and NLR function is the NFκB pathway [96,97]. NFκB can serve as either an upstream (e.g., NLRP3) [94] or downstream (e.g., TLR) [98] signaling pathway and can even function as both, leading to positive feedback loops (e.g., NOD2) [99]. In quiescent cells, NFκB (e.g., p65/p50) is constrained within the cytoplasm by binding to an inhibitory protein, IκB. Detection of PAMPs by PRRs activates IκB kinase which phosphorylates IκB licensing it for dissociation from NFκB and targeting it for proteasomal degradation. The freed NFκB translocates from the cytosol to the nucleus, where it transactivates relevant genes. The NFκB-induced antimicrobial secretome includes mucins, antimicrobials, as well as chemokines to recruit circulating phagocytes [96,100,101]. As a negative feedback control mechanism, NFκB also enhances IκB synthesis to silence NFκB activity.

Predictably, epithelial and/or macrophage TLRs [102,103], NLRs [104,105,106,107,108], their recognition receptors (PRRs), and downstream effectors such as NFκB or Il-1β [96,108,109,110] have been implicated in both experimental and clinical IBD and ARDS. 

## 5. Transduction of Microbial Signals to an Antimicrobial Response: Vitamin D/VDR Signaling

VDR can modulate microbial sensing (e.g., PRRs) and effector responses (e.g., NFκB) by both transcriptional-dependent and transcriptional-independent mechanisms [111]. Direct evidence for an impact of vitamin D/VDR signaling on PRRs or NFκB pathways within the epithelium and/or resident macrophages of either the gut or lungs is limited [112]. Relevant issues have been addressed primarily in more tractable cells, such as epithelial-like cell lines, keratinocytes, fibroblasts, macrophage-like cell lines, and monocytes. Rather than exhaustive documentation of these studies, several salient features will be highlighted that underscore the complexity of the VDR modulation of sensor and effector pathways. 

The role of vitamin D/VDR signaling in TLRs appears to be cell- and context-specific. In isolated human monocytes, vitamin D/VDR signaling downregulates both message and protein levels of plasma membrane TLR2 and TLR4 [113,114]. The decreased expression of these TLRs is associated with functional consequences, e.g., decreased nuclear translocation of NFκB [113]. In a similar cell system, vitamin D/VDR signaling differentially impacts the endosomal membrane of TLRs; TLR 9 expression is decreased, while TLR3 and TLR7 levels are not affected [114]. Again, the decrease in TLR9 expression impacts downstream outcomes, i.e., there is a decrease in interleukin 6 production. Of note, despite the vitamin D/VDR-mediated decrease in TLR2 and TLR4, CD14 a co-receptor and promoter of TLR2 and TLR4 function, is upregulated. This may be partially explained by the fact that CD14 and TLR4 can sometimes function independently [115]. In skin injury models, vitamin D/VDR signaling increases both CD14 and TLR2 in keratinocytes [116]. Since exogenous TGF-β can mimic this response, the vitamin D/VDR- mediated upregulation of both TLR2 and CD14 may be a component of a post-injury healing process. 

The available data from epithelial and monocytic cell lines indicate that the cytosolic PRRs, NOD2 and NLRP3, are differentially modulated by the vitamin D/VDR signaling pathway. In human cells, the study by Dimitrov et al. [117] identified several thousand distinct genes up- or downregulated by 1, 25D or its analogs in a highly cell-specific manner using a 1.5-fold cut-off. This included cell-specific regulation of several components of NOD-like pattern-recognition receptor signaling. The NOD2 gene contains VDREs in distal enhancer elements and vitamin D/VDR signaling increases cytosolic levels of NOD2 as well as the downstream NFκB activity [118]. By contrast, the bulk of the evidence indicates that the vitamin D/VDR pathway inhibits NLRP3 inflammasome assembly and function [18,119]. The mechanisms include both direct and indirect effects of VDR. The direct binding of VDR to NLRP3 prevents its posttranslational de-ubiquitination and thereby its oligomerization and generation of active IL-1β [120]. The binding of VDR to NLRP3 requires the C-terminal LBD, but not the N-terminal DBD, of VDR and can prevent inflammasome formation/activation within 30 min, a response time negating a transcriptional event. In addition, vitamin D/VDR inhibits numerous priming and activating signals upstream to inflammasome assembly and activation, e.g., VDR dampens ROS [121,122] and NFκB (see below). There may be a context-dependent opposite effect of vitamin D/VDR signaling on Il-1β generation by the NLRP3 inflammasome. Specifically, the vitamin D/VDR pathway promotes IL-1β secretion by tuberculosis-infected macrophage-like cells, but not by uninfected cells [123]. 

The NFκB effector pathway is impacted by VDR at multiple levels, including transcriptional and post-transcriptional mechanisms. As compared to wild-type fibroblasts, the basal NFκB activity of VDR^−/−^ fibroblasts is elevated; a response is attributed to lower cytosolic levels of IκB, most likely due to increased ubiquitination and proteasomal degradation [124,125]. The transcriptional complex of VDR/RXR can bind the p65, but not p50, subunit of NFκB and can prevent either VDR-mediated or p65-mediated transcription [126,127]. The DBD of VDR is required for the transcriptional repression of an NFκB-inducible gene (p40 subunit of IL-12); deletion of the NFκB binding site from the p40 promoter abrogated the repression [126]. 

Vitamin D/VDR signaling can also modulate the NFκB pathway by post-transcriptional mechanisms. VDR can inhibit LPS or TNF-α stimulated NFκB activation by binding to the IκB kinase [18,128]. The binding of VDR to the IκB kinase results in the stabilization of IκB and inhibition of NFκB activation. The binding of VDR to IκB kinase requires the LBD, but not the DBD of VDR, and effects are apparent within a time frame too short for a transcriptional event. Further, VDR can bind to the p65, but not p50, subunit of NFκB; the VDR/p65 complex does not enter the nucleus but remains confined to the cytosol of isolated colonic epithelial cells [112]. The latter observations support a physical interaction between VDR and NFκB specific for the p65 subunit and independent of transcriptional events. As was the case with VDR^−/−^ fibroblasts [124], VDR^−/−^ epithelial cells had decreased levels of IκB and increased nuclear p65 [112]. Of note, in the absence of vitamin D, *S. typhimurium* infection of epithelial cells was sufficient to increase VDR protein expression and subsequent transcriptional activity. While the specific mechanisms involved are not clear, VDR activation by non-vitamin D ligands is not unprecedented; cholesterol derivatives (e.g., bile acids) can activate VDR [17]. 

The role of vitamin D status on microbial infection and associated sequelae appears to differ between the gut and lungs. VDR^−/−^ mice are more susceptible to colonic inflammation induced by *S. typhimurium* infection [112]. In line with this, dietary vitamin D deficiency increases the susceptibility of mice to colitis induced by *C. rodentium* infection [129]. A clinical study of patients infected with *C. difficile* indicates that disease severity is inversely related to circulating levels of 25(OH)D [130]. By contrast, the inflammatory response in the lung to *P. aeruginosa* or *S. pneumoniae* infection is not impacted by diet-induced vitamin D deficiency [131]. Further, clinical trials assessing the effects of vitamin D supplementation on the risk of all-cause respiratory tract infections or COVID-19 indicated a modest, if any, benefit [50,132]. Interestingly, supplementation of IBD patients with vitamin D had no effect on the incidence of influenza but did reduce the incidence of upper respiratory tract infections [133]. It is important to mention that the severity of IBD worsened in the ulcerative colitis subgroup who received vitamin D supplementation, but this effect was minimal [133]. Despite these limitations, it has been proposed that daily vitamin D supplementation may improve resistance to respiratory infections [50,134].

## 6. Epithelial Secretion of Mucus 

The viscoelastic property of mucus can impede the mobility of microbes and thus restrict their access to the epithelium. However, this same property would also hinder nutrient absorption in the gut and gas exchange in the lungs. Thus, spatial segregation of the mucus barrier is required for the proper functioning of both intestines and lungs. For example, the extensive and dense mucus covering the colonic epithelium hinders encroachment of the epithelium by the microbiota, while the rather sparse mucus of the upper small intestine facilitates nutrient absorption [4,5,135,136]. An analogous situation exists in the lungs. A mucous layer is present in the bronchi to entrap microbes and limit their distribution downstream, but mucus is absent in the alveoli where gas exchanges occur [137,138].

The epithelium of both gut and lungs synthesizes mucin glycoproteins that contain a transmembrane domain (e.g., MUC1, MUC4, MUC16) which form the glycocalyx [5,6]. MUC1 contains both an ectodomain and a cytoplasmic domain. An interesting feature of MUC1 is its ability to thwart microbe encroachment of the epithelium (via ectodomain shedding) and inhibit inflammatory responses (via intracellular signaling) [139]. Bacterial infection/injury of the gut is exaggerated in MUC1^−/−^ mice [140]. MUC1 also limits viral and bacterial respiratory infections [141,142]. 

The epithelium of the gut and lungs also secrete mucus; the specific mucin glycoproteins differ between the two organs. MUC2 mucin is secreted by small and large intestinal goblet cells [69,143]. The mucus coating the small intestine is sparsely facilitating nutrient assimilation [135,136], while that of the colon is abundant and serves as a microbial habitat [4,143]. MUC5AC and MUC5B mucins are secreted by goblet cells of collagenous bronchi and club cells of distal respiratory bronchi [72,138,144]. MUC5B is the dominant mucin of the distal bronchioles, while the alveoli are lined by a thin surfactant layer rather than mucus [6,72,144]. Further, the alignment of the mucous layer relative to the epithelium is strikingly different between the colon and bronchi. 

Colonic mucus is described as a continuum of a compact gel-like layer adjacent to the epithelium which gives rise to a loose sol-like layer blending with luminal contents [78,143]. The nutrient-rich, sol-like layer provides a habitat for commensals [4,69,143]. The more gel-like layer adjacent to the epithelium is selectively permeable, restricting access of bacteria to the crypts while allowing smaller molecules (e.g., SCFA) to access the surface epithelium [145]. The commensals of the proximal colon induce copious mucus secretion which segregates the microbes to the central stream of undigested material [146,147]. This microbe-mucus dynamic is beneficial for both microbes and hosts (symbiotic). Diversion of microbes from the epithelium prevents infection and sequestering microbiota with a nutrient source (e.g., undigested carbohydrates) generates metabolites (e.g., SCFA) that are used by colonocytes [26]. The mucus/bacteria-laden digesta is dehydrated as it proceeds to the distal colon, forming feces. The shear stress induced with the passage of hardened feces removes a significant fraction of the gel-like mucus from the epithelium, all of which is eliminated during defecation [146,147,148]. The mucus lost is replaced by additional secretion from goblet cells (the mucus turnover being on the order of hours) [135]. MUC2^−/−^ mice spontaneously develop colitis, indicating that colonic mucus serves an important protective function [149,150]. Further, in colonic biopsies from IBD patients, MUC2 secretion by goblet cells was defective, MUC2 level in the mucous layer adjacent to the epithelium was reduced, and the normally impenetrable inner gel-like layer was permeable to bacteria, even in uninflamed areas [145,151,152]. The latter observations argue against the mucus defect being a result of the disease but favor a causative role, i.e., an aberrant mucous layer being a risk factor for IBD. 

Unlike the case in the colon where the gel-like mucous layer abuts the epithelium transitioning to a sol-like layer that mixes with lumen contents, the gel-like and sol-like mucous layers in the bronchioles are inverted. A sol-like periciliary layer is adjacent to the bronchial epithelium, while a gel-like layer floats above it [137,138]. This arrangement allows cilia projecting from epithelial cells to move freely. The metachronal waves generated by the cilia within the periciliary layer propel the overlying mucus (and any entrapped microbes) back towards the upper airways for eventual elimination, i.e., mucociliary clearance (MCC) [138]. Mucin lost because of MCC is replenished by bronchial goblet cells within hours; the mucus turnover rate in the lungs is like that of the gut. Predictably, the gel-like properties of bronchial mucus can impede the diffusion of microbes; diffusivity is inversely proportional to gel density [153]. However, unlike the situation in the gut, an increase in mucus density can more readily increase microbial infections [154]. An increase in mucin concentration or a decrease in mucus hydration impairs ciliary movement and MCC [155]. MUC5B mucins appear to be more important in this regard than the MUC5AC mucins. MCC is decreased in MUC5B^−/−^ mice, but not in MUC5AC^−/−^ mice and the MUC5B^−/−^ mice are more susceptible to bacterial infection [156]. 

## 7. Epithelial Secretion of Mucus: Vitamin D/VDR Signaling 

There is a paucity of information on the regulation of either lung or gut mucus secretion by vitamin D/VDR signaling. While bronchial and alveolar epithelial cells express VDR [34,39], whether bronchiole goblet/club cells express VDR or respond to vitamin D has not been addressed. There are two lines of evidence, albeit tenuous, implicating vitamin D/VDR signaling in colonic mucus secretion. However, since intestinal goblet cells do not express VDR [31,32,33], a direct effect of vitamin D/VDR signaling in goblet cells seems unlikely and alternative mechanisms have been posited. First, goblet cell morphology is aberrant in either pan- or epithelial-specific VDR^−/−^ mice, an effect attributed to low systemic calcium levels [33,157]. Second, the colonic mucous layer is thinner in CYP27B1^−/−^ mice, an effect attributed to an outgrowth of mucin-degrading microbes [158]. As addressed previously, microbes do not express VDR, and thus the dysbiosis noted in CYP27B1^−/−^ mice is most likely due to vitamin D/VDR signaling in epithelial or myeloid cells of the host. 

**Figure 1:**
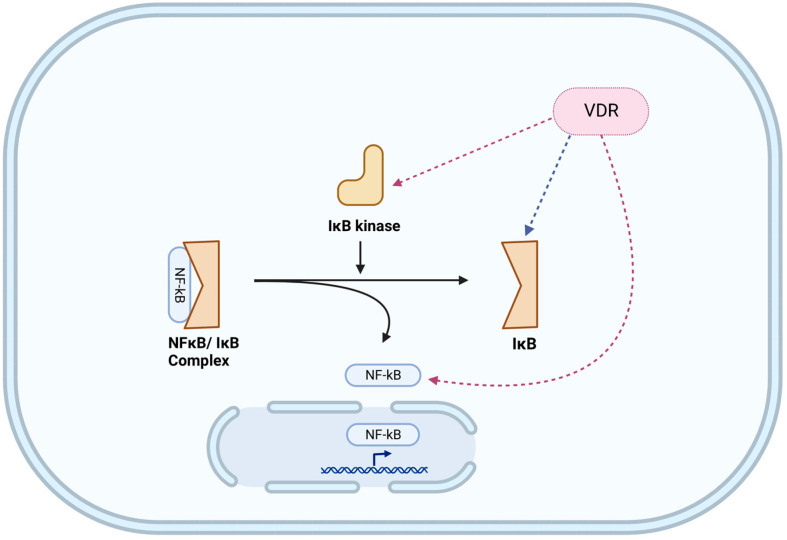
Impact of vitamin D/VDR signaling on modulating the NFκB pathway. Experimentally verified inhibitory pathways are indicated with red dashed arrows, while an ambiguous pathway is indicated with a blue dashed arrow. The NFκB effector pathway is impacted by VDR at multiple levels, including post-transcriptional mechanisms. Figure was created with BioRender.com (accessed on 4 March 2023).

The lack of vitamin D/VDR signaling in goblet cells may be fortuitous. In response to microbes (or their products), goblet cells elicit copious mucus secretion to repel microbes from the epithelial surface. The promoter regions of intestinal *MUC2* [100] as well as bronchial *MUC5B* [159] and *MUC5AC* [160] contain binding motifs for NFκB, a major transcription factor involved in host defense against infection. Since VDR inhibits the NFκB pathway at multiple points (Figure 1), inhibition of these pathways by vitamin D/VDR signaling would not be of benefit to the host. 

## 8. Epithelial Secretion of Antimicrobial Peptides

The epithelium of both organs plays a crucial role in host defense by secreting antimicrobial peptides (AMPs). These amphipathic cationic substances disrupt microbial membranes and are particularly important in regions of the gut and lungs that lack a significant mucus barrier, such as the small intestine and alveoli, respectively [101,161]. In regions with a mucus coating, the secreted AMPs are retained within the mucus, providing a combined physical and chemical barrier against microbial infection [162]. Specialized Paneth cells found in the crypts of Lieberkühn secrete AMPs in the intestines [30], whereas in the lungs, AMPs are secreted into the airway surface liquid (ASL), a thin layer of fluid rich in mucin, and are produced by the submucosal glands of the respiratory epithelium [19,163,164,165].

## 9. Epithelial Secretion of Antimicrobial Peptides: Vitamin D/VDR Signaling 

Vitamin D plays a critical role in the host’s defense against bacterial, fungal, and viral infections by stimulating the production of AMPs, which act as endogenous antibiotics and are among the body’s first lines of defense [19,166,167,168,169].

In human epithelial cells, vitamin D/VDR signaling directly induces the transcription of genes that encode AMPs. Two main AMPs are transcribed from human cathelicidin antimicrobial peptide (*CAMP*) and human β-defensin 2/defensin-β4 (*HBD2*/*DEFB4*) [19]. The VDR binds to vitamin D response elements on the proximal *CAMP* and *HBD2* promoter sequences [19,30,170,171], located 507 and 1231 bp upstream of their transcription initiation sites, respectively [172]. While these genes are induced by vitamin D in human cells of epithelial or myeloid origin, the same process does not seem to occur in cells derived from mice [19,173]. An intriguing observation to consider is that in human peritoneal macrophages, vitamin D induced the expression of *CAMP* but suppressed the expression of hepcidin antimicrobial peptide (*HAMP*) [173]. 

An interesting evolutionary distinction is that VDR induction of *CAMP* does not occur in mice. This may be attributed to the fact that the vitamin D response element (VDRE) is absent in murine, rat, and canine *CAMP* promoters, but conserved in primates, such as humans and chimpanzees [19,170,171,174]. According to the research by Gombart et al. [174], the VDRE located in the human *CAMP* promoter is incorporated within an Alu repeat transposable element that is unique to humans and primates and seems to have been inserted prior to the separation of Old World and New World primates, as the VDRE appears to be highly functionally and structurally conserved across all primates [19,174]. Experiments have shown that exposure of murine cells to vitamin D did not promote antibacterial activity, indicating that this process appears to be inherently found in primates [19,173].

Vitamin D/VDR signaling leads to an upregulation in the expression of *CAMP*, which ultimately leads to the production of hCAP-18. hCAP-18 is a propeptide that consists of an N-terminal cathelin domain which functions as a cysteine protease inhibitor that has a broad-spectrum antibacterial action and a C-terminal LL-37 domain [166,170]. The LL-37 domain is cleaved off by serine proteases from the kallikrein family [169] and exhibits specific antibacterial, antimycotic, and antiviral activity against *Mycobacterium tuberculosis* [166,167], *Candida albicans* [166,168,175], and HIV [19,166,175], respectively, as mentioned in the study by Meyer et al. [166] and antibacterial activity against *Chlamydia trachomatis*, as described by Mabrouk [175]. 

The antibacterial properties of LL-37 and *HBD2* are attributed to their ability to disrupt bacterial cell membranes through interaction with their hydrophobic and phospholipid constituents [19,170,176]. LL-37, the only human cathelicidin [177], exists as a cation with no specific secondary structure in solutions but acquires an amphipathic α-helical structure upon entry into bacterial cell membranes [170,176]. This enables LL-37 to bind to specific bacterial components such as lipopolysaccharide and lipoteichoic acid in Gram-negative and Gram-positive bacteria, respectively, thereby inhibiting TLR signaling and subsequent bacterial inflammation [176]. Additionally, LL-37 can stimulate respiratory epithelial cells by transactivating the epidermal growth factor receptors (EGFRs) [169,177,178], which was shown to enhance wound healing by keratinocytes by releasing heparin-binding-EGF (HB-EGF) that interacts with EGFR [170].

According to a study conducted by d’Aldebert et al. [179], in biliary epithelial cells, bile salts play a crucial role in maintaining a sterile environment by signaling through the VDR and farnesoid X receptor (FXR), which regulate the expression of *CAMP*. 

In the study conducted by Rosa et al. [30], mice fed vitamin D-deficient diets had increased colonic expression of proinflammatory markers and β-defensins, and a decreased expression of AMPs such as ileal α-defensin 5, lysozymes, and cryptdins. Examination of the Wingless and Int (Wnt) signaling molecules Wnt3, Wnt5a, and Wnt9a in the ileum revealed a reduction in expression of Wnt5a in the mice fed a vitamin D-deficient diet when compared to the control. Rosa et al. [30] sought to test their hypothesis that, in addition to the Wnt signaling pathway, vitamin D regulates gene expression through the mitogen-activated protein kinase (MAPK) and Janus kinase (Jak)/signal transducers and activators of transcription (STAT) pathways. By using the organoid cell model, vitamin D was shown to increase AMP mRNA expression via the Jak/STAT 5 pathway, but the MAPK pathway was not shown to be involved in the vitamin D/VDR-gene regulation [30]. However, data in another study by Guo et al. [180] revealed that vitamin D can modify gene expression through the p-38-MAPK pathway in renal tubular epithelial cells, albeit the end result is not relevant to the modulation of AMPs, as VDR activation inhibited p-38-MAPK signaling, resulting in decreased tubular epithelial cell apoptosis in diabetic nephropathy [180]. The study by Rosa et al. [30] revealed that vitamin D/VDR allowed the exposure of binding sites for p-STAT5, resulting in the formation of VDR/STAT5 complexes that allow for the induction of anti-inflammatory cytokines. It was therefore illustrated that vitamin D/VDR regulates intestinal AMP expression through both Wnt and Jak/STAT5 signaling pathways [30].

AMPs have been also recently implicated in the antiviral innate response as well, with studies generated based on experiments mostly carried out in vitro for viruses other than the extensively researched HIV [19,166,175,181,182]. For example, in the in vitro experiments performed by Telcian et al. [37], human bronchial epithelial cells were exposed to the respiratory syncytial virus (RSV) and rhinovirus, and the study demonstrated that exogenous administration of vitamin D resulted in reduced replication and release of rhinovirus and revealed cathelicidin’s direct action against rhinovirus [19,37]. In RSV, infection of bronchial epithelial cells increased 1 α-hydroxylase mRNA expression and significantly increased cathelicidin mRNA [19,37]. Currie et al. [183] showed that increased *CAMP* expression by vitamin D protects against epithelial cell RSV-induced apoptosis as well as suppressed RSV replication and viral particle assembly [19,183].

Another mechanism by which LL-37 and other AMPs aid in the innate antiviral immune response is by directly disrupting the viral envelope, most notably demonstrated in the influenza A virus and RSV, as shown in the review by White [19]. In the case of the influenza A virus, LL-37 was shown to interact with the virus’s InA capsids, thereby damaging viral membrane integrity, a unique mechanism when compared to other innate inhibitors such as human neutrophil defensins (HNPs) and surfactant protein D (SP-D) [19,177]. Again, in the experiment by Currie et al. [183], LL-37 was also shown to interact with RSV, thereby destroying the virus’s envelope [19,183]. 

It has been shown that vitamin D-induced AMPs enhance TLR signaling. For example, LL-37, which is a positively charged particle, binds to the negatively charged nucleic acids that comprise the viral genome, thus enhancing their endocytosis and detection by TLRs, specifically TLR3, TLR7, or TLR9, thereby resulting in an increased level of circulating interferons [19,184,185].

The antimicrobial activity in airway surface liquid could be affected by seasonal variation. A randomized placebo-controlled trial by Vargas et al. [186] aimed to study the seasonal antimicrobial activity of ASL and how it could be affected by vitamin D levels. The study showed that the ASL’s antimicrobial activity is lower in winter–spring seasons compared to summer–fall seasons and that adding an LL-37 neutralizing antibody suppressed the ASL’s antimicrobial activity from summer to fall [19,186]. Moreover, a previous study by the same group was carried out in 2017 [187] and showed that antimicrobial activity may be affected by serum vitamin D3 levels which also followed a seasonal pattern.

In the case of SARS-CoV-2, the cause of the global COVID-19 pandemic, the role vitamin D plays in the secretion of AMPs is of particular interest currently, and research is being heavily generated in this field [175,188]. In silico studies showed that LL-37 [189,190] and *HBD-2* [191] can bind to the virion’s receptor binding domain (RBD) found on its spike proteins and conceal its cellular receptor, ACE2, therefore blocking viral entry [19,188,189,190,191]. Research is still ongoing, and many papers have yet to be peer-reviewed, but indeed prove to be promising, as also portrayed in White’s review [19].

## 10. Conclusions 

In conclusion, our review emphasizes the crucial role of vitamin D in regulating the innate immune response in the lungs and intestines and highlights the potential of vitamin D supplementation in managing various infectious diseases while maintaining the homeostasis of the pulmonary and intestinal epithelial barriers. However, further studies involving human subjects or primary cell lines are necessary to provide more robust evidence supporting the mechanisms underlying the observed effects of vitamin D on the immune response. Therefore, randomized controlled trials that nest mechanistic outcomes within are necessary to investigate the potential of vitamin D as an adjunct in the management of infectious diseases of the pulmonary and gastrointestinal systems, as well as in inflammatory bowel disease and acute respiratory distress syndrome. Moreover, age is a crucial factor that should be taken into consideration when investigating the potential benefits of vitamin D supplementation in infectious diseases. This review provides insights into the immunoregulatory properties of vitamin D and its potential role in promoting immune homeostasis and may stimulate further research in this field.

## Figures and Tables

**Table 1:** Distribution of VDR, CYP27B1, and CYP24A1 in the epithelium of the gut and the lung. +: present, -: absent, ?: no data available.

Cell Type	VDR	CYP27B1	CYP24A1	References
Gut				
Enterocyte	+	+	+	[21,22,23,24]
Colonocyte	+	+	+	[21,23,25,26,27]
Paneth cells	+	?	?	[26,28,29,30]
Goblet cells	-	?	-	[21,22,26,31,32,33]
Lungs				
Bronchial epithelium	+	+	+	[21,34,35,36,37,38]
Alveolar type II cells	+	+	+	[39,40]
Alveolar type I cells	?	?	?	

## Data Availability

Not applicable.
